# Notes and News

**Published:** 1901-06-08

**Authors:** 


### Notes and News.

Amongst the Victoria Crosses most recently awarded is one to Captain N. R. House, New South Wales Medical Corps, who rescued a wounded soldier under heavy fire at Vredefort in July, 1900.

Amongst the 22,000 recruits rejected from the Army in 1899 upwards of one thousand were rejected as suffering from disease of the heart. This seems a very large percentage of sufferers from so serious a disease, especially as those rejected for loss of or decay of teeth only exceeded the number by seven hundred.

We are now nearly half through the year 1901, and London still waits for a reasonable amount of accommodation for dealing with female inebriates under the Act passed in 1898. A Visiting Justice writes to the *Times* from Holloway Prison, saying that there are now over 100 women in the prison qualified for an inebriate home, always in and out, and always a trouble to everyone connected with the prison. Yet the London County Council home still lingers on the stocks.

The authorities of the Worcester Infirmary have resolved to close the Garlick Ward as a means of reducing the expenditure of the infirmary, which exceeds its income. As we pointed out before, the measure is a drastic one; if the accommodation is really in demand it injures a large number and saves but little. On the other hand, if the authorities feel that the infirmary can meet the requirements of the district without the ward, the action is judicious, and in any case may stimulate the population of Worcester and the district to contribute enough to keep the infirmary out of debt. The diminution in the staff will be two probationers and one wardmaid. All the probationers at the Worcester Infirmary pay a premium, and no salary, or one of £5 or £10, is given. It is also stated that the infirmary will not require an assistant matron, who otherwise would have been necessary, though one has not hitherto been employed. The saving to the infirmary is thus made to assume the largest proportions possible.

Every year for 23 years an anonymous donor with the *nom de plume* of "Delta" has sent a contribution of £200 to the Hospital Sunday Fund.

The plague still claims fresh victims at the Cape. Up to the present time 318 deaths have occurred, and upwards of 680 persons have been attacked. It is also present in Egypt, but the cases are few.

The new missionary steamer *Livingstone*, built for the Congo-Balolo Mission, contains a fully-equipped hospital department. The ship is fitted with mosquito-proof gauze panels to reduce the risk of malarial fever. A dispensary has also been provided.

A cottage hospital has been opened at Blairgowrie by Sir James Clerk-Rattray, K.C.B. Mrs. Macpherson, of Blairgowrie, gave the site, Sir J. Clerk-Rattray gave £1,000, and other generous friends provided a sum of upwards of £3,400. The hospital, with furnishing and equipment, cost £2,594. It provides accommodation for six inmates.

At the annual meeting of the Hospital for Consumption, Brompton, it was stated that the adoption of the open-air treatment in one portion of the building had proved most successful. A country branch and convalescent home is to be erected at Heatherside, near Bagshot. This should prove a valuable addition to the most useful work already done by the hospital.

At the annual meeting of the National Society for the Employment of Epileptics, held on Monday, it was stated that further accommodation was needed at the Colony at Chalfont St. Peters to meet the demands; and that, as a result of the treatment at the Homes, many ex-colonists, both men and women, are now supporting themselves by their own exertions in the outside world.

Lord Lister, F.R.S., will open the new operating theatre and children's ward at St. Thomas's Hospital on June 20th at 4 p.m. The theatres have been fitted with all the latest improvements for the carrying out of aseptic surgery, and the new block of buildings is provided with an artificial system of ventilation. The children's ward is elaborately decorated with pictorial representations of fairy tales and nursery rhymes in tiles, the expense being defrayed by one of the governors of the hospital.

Madame Rosa Bird, who is giving an Australian Concert under distinguished patronage for the benefit of the Colonial Troops Entertainment Fund at Steinway Hall at 3 o'clock on Monday, June 10th, has most generously placed a number of seats at the disposal of the members of the colonial contingents and invalided soldiers who are in this country. Among others who are taking part in this concert may be mentioned Miss Regina Nagel, who in Australia sang "Ben Bolt" in *Trilby*, and Mr. Arthur Deane, baritone, of the Carl Rosa Opera Company. Members of the Colonial Corps who desire to avail themselves of Madame Rosa Bird's liberal offer of gratuitous seats should apply immediately to their respective colonial agents or to the secretary of the Colonial Troops Entertainment Fund, 71 and 72 King William Street, E.C., when orders for admission (unless previously exhausted) will be forwarded.

